# Strong hydrogen bonding in a dense hydrous magnesium silicate discovered by neutron Laue diffraction

**DOI:** 10.1107/S2052252520003036

**Published:** 2020-04-02

**Authors:** Narangoo Purevjav, Takuo Okuchi, Christina Hoffmann

**Affiliations:** a Institute for Planetary Materials, Okayama University, 827 Yamada, Misasa, Tottori 682-0193, Japan; b Neutron Scattering Division, Neutron Sciences Directorate, Oak Ridge National Laboratory, Tennesee, TN 37831, USA

**Keywords:** hydrogen bonding, Earth’s deep mantle, dense hydrous magnesium silicates, neutron diffraction

## Abstract

A large amount of hydrogen circulates inside the Earth, which affects the long-term evolution of the planet. The majority of this hydrogen is stored in deep Earth within the crystal structures of dense minerals that are thermodynamically stable at high pressures and temperatures. To understand the reason for their stability under such extreme conditions, the chemical bonding geometry and cation exchange mechanism for including hydrogen were analyzed in a representative structure of such minerals (*i.e.* phase E of dense hydrous magnesium silicate) by using time-of-flight single-crystal neutron Laue diffraction. Phase E has a layered structure belonging to the space group *R* 3 *m* and a very large hydrogen capacity (up to 18% H_2_O weight fraction). It is stable at pressures of 13–18 GPa and temperatures of up to at least 1573 K. Deuterated high-quality crystals with the chemical formula Mg_2.28_Si_1.32_D_2.15_O_6_ were synthesized under the relevant high-pressure and high-temperature conditions. The nuclear density distribution obtained by neutron diffraction indicated that the O—D dipoles were directed towards neighboring O^2−^ ions to form strong interlayer hydrogen bonds. This bonding plays a crucial role in stabilizing hydrogen within the mineral structure under such high-pressure and high-temperature conditions. It is considered that cation exchange occurs among Mg^2+^, D^+^ and Si^4+^ within this structure, making the hydrogen capacity flexible.

## Abstract 2

By using neutron Laue diffraction, strong hydrogen bonding was observed in the framework structure of phase E of dense hydrous magnesium silicate. Such bonding plays a crucial role in stabilizing large amounts of hydrogen in the crystal structures of minerals under high-pressure and high-temperature conditions inside deep Earth.

## 1. Introduction   

**Figure 1:**
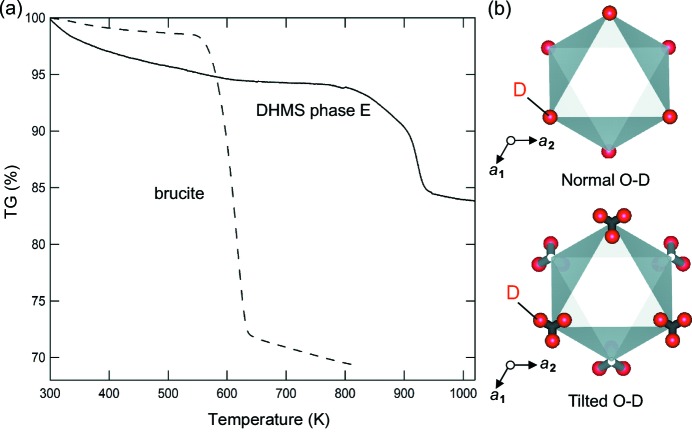
(*a*) Thermogravimetry results of DHMS phase E [Mg_3−0.5*x*_Si*_x_*H_6−3*x*_O_6_] and brucite [Mg(OH)_2_] measured at ambient pressure using Rigaku Thermo plus EV02. The former structure retains most of its hydrogen at around 900 K, while the latter retains it at around 600 K. We consider that the difference in dehydration temperatures between these minerals is related to the difference in their hydrogen bonding strengths. (*b*) Two equally plausible hydrogen site models that have been adopted so far for DHMS phase E (Tomioka *et al.*, 2016 ▸). Each corner of the octahedra is made of an oxygen anion (not shown). In the normal O—D model, the dipole is parallel to the *c* axis and normal to the MgO_6_ octahedral layers. In the tilted O—D model, the dipole is tilted from the *c* axis. The crystallographic illustrations were created using the software *VESTA3* (Momma & Izumi, 2011 ▸).

Hydrogen can be incorporated into minerals in highly variable amounts. Once incorporated, the hydrogen is circulated throughout the Earth, from the surface to the deep interior, affecting the long-term evolution of the planet (Iizuka-Oku *et al.*, 2017 ▸; Kawakatsu & Watada, 2007 ▸; Okuchi, 1997 ▸; Thompson, 1992 ▸). A major proportion of this hydrogen is currently stored within the crystal structures of dense minerals that are thermodynamically stable under the high pressures and temperatures of the deep mantle of the Earth (Ohtani, 2015 ▸; Purevjav *et al.*, 2014 ▸, 2016 ▸, 2018 ▸; Sano-Furukawa *et al.*, 2018 ▸). Dense hydrous magnesium silicates (DHMSs) are the most typical among such dense mineral species; they have very large hydrogen capacities even under extreme pressure and temperature conditions (Frost, 1999 ▸; Nishi *et al.*, 2014 ▸; Ohtani *et al.*, 2000 ▸). Phase E [Mg_3−0.5*x*_Si*_x_*H_6−3*x*_O_6_] has the largest hydrogen capacity (18% H_2_O weight fraction of the total mass) and one of the best thermodynamic stabilities among DHMSs; it is stable to temperatures of at least 1573 K and pressures of 13–18 GPa (Kanzaki, 1991 ▸; Frost, 1999 ▸). Fig. 1 ▸(*a*) shows the thermogravimetry result of DHMS phase E synthesized under high-pressure and high-temperature conditions. Most of the hydrogen was retained inside the crystal structure up to 900 K under ambient pressure, which is distinctly higher than that typically seen for common hydrogen-bearing minerals of lower density. To understand the reason for such high-temperature stability of hydrogen in the mineral structure, which fundamentally controls the circulation of hydrogen within deep Earth, its chemical bonding geometry and cation-exchange mechanisms must be fully clarified.

X-ray diffraction analysis has been used to examine the framework of DHMS phase E without hydrogen. It was revealed to have a layered structure belonging to a trigonal crystal system (space group 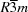) (Kudoh *et al.*, 1993 ▸). The structure consists of two different magnesium sites (Mg1 and Mg2), one silicon site (Si) and one oxygen site (O). Each Mg^2+^ ion is surrounded by six O^2−^ ions to form MgO_6_ octahedra, and each Si^4+^ ion is connected to four O^2−^ ions to form SiO_4_ tetrahedra. Most of the Mg^2+^ ions are located at the Mg1 sites, which collectively form a layer of edge-sharing MgO_6_ octahedra. However, there was also a minor amount of Mg^2+^ occupying the Mg2 sites outside this layer. The SiO_4_ tetrahedra are distributed statistically between two adjacent MgO_6_ layers together with the possible hydrogen sites; however, hydrogen was not detectable using X-ray diffraction.

In order to locate the hydrogen sites, we previously analyzed the structure of deuterated DHMS phase E using powder neutron diffraction at J-PARC, Japan (Tomioka *et al.*, 2016 ▸). Two equally plausible hydrogen site models (normal and tilted O—D dipole models) were derived [Fig. 1 ▸(*b*)]. The hydrogen concentrations determined based on the two models were very similar, derived from their refined site occupancies. Thus, we concluded that the hydrogen concentration within the mineral structure was reasonably constrained, where the refined site occupancies of hydrogen were compatible with the mineral stoichiometry. On the other hand, the powder data did not allow us to discriminate between the geometries of the hydrogen bonds among these models, owing to the insufficient spatial resolution. Thus, in this study, we employed time-of-flight (TOF) single-crystal neutron Laue diffraction for our synthesized high-quality DHMS phase E crystal. To determine the most accurate bonding distances of hydrogen, we synthesized a fully deuterated crystal. We expect that the heavier mass of deuterium relative to protium should reduce its vibration/displacement at its equivalent sites. In addition, the longer coherent scattering length of deuterium relative to protium should help to increase the signal-to-noise ratio, and its shorter incoherent scattering length should reduce background scattering. Hence, to obtain the best possible dataset given the very small crystal size, we used a fully deuterated sample to reduce the background and increase the signal-to-noise ratio. Therefore, the TOF Laue scheme will allow very high sensitivity for detecting weaker reflections at lower *d*-spacings from a small synthetic crystal. In our previous studies conducted using this combination, reflections with minimum *d*-spacings (*d*_min_) as low as 0.3 Å were successfully resolved and analyzed (Purevjav *et al.*, 2016 ▸, 2018 ▸), enabling quantitative determination of site positions and occupancies of deuterium in DHMS phase E.

## 2. Materials and methodology   

### 2.1. Single-crystal synthesis and characterization   

Fully deuterated single crystals of DHMS phase E were synthesized under high-pressure and high-temperature conditions using a scaled-up Kawai-type cell. We previously established a slow-cooling method for growing physically and chemically homogenous crystals of hydrogenated minerals that exist in deep Earth (Okuchi *et al.*, 2015 ▸). This method proved applicable for preparing the deuterated crystals. A mixture of Mg(OD)_2_ and SiO_2_ powders at a 2:1 molar ratio was used as the starting material. The Mg(OD)_2_ was synthesized from dried MgO powder and D_2_O water in an autoclave at 513 K and 40 MPa. Raman spectroscopy confirmed that the Mg(OD)_2_ had no hydrogen contamination (Okuchi *et al.*, 2014 ▸). The SiO_2_ powder was prepared from a high-purity glass rod; the glass contained less than 20 p.p.m. OH groups. The mixture was sealed in a gold sample capsule (4 mm outer diameter and 4.5 mm length). The capsule was placed in an 18/10 type Kawai cell. To synthesize a fully deuterated crystal, we prebaked the cell parts at 1273 K for 1 h before the synthesis experiment to completely remove any absorbed hydrogen. The cell was combined with eight tungsten carbide anvils in a dry environment (laboratory humidity <40%), which had edge lengths of 46 mm. The sealed cell was compressed to a pressure of 15 GPa; then it was heated to 1366 K and slowly cooled to 1348 K over 3 h to grow the crystals. Subsequently, the cell was quenched rapidly to room temperature by cutting off the heater power. Finally, the pressure was released and the grown crystals were recovered under ambient conditions. Many crystals with the same composition grew together within the capsule, and were confirmed to have the DHMS phase E structure by X-ray diffraction analysis. We carefully selected one of the largest crystals for neutron diffraction, with a volume of 0.1 mm^3^ (0.65 × 0.5 × 0.3 mm). The crystal was optically transparent, *i.e.* there was an absence of inclusions, twinning and cracks when observed under a polarized optical microscope (Fig. S1).

### 2.2. Time-of-flight single-crystal neutron Laue diffraction   

The selected sample crystal was studied using the TOPAZ diffractometer installed at Spallation Neutron Source, Oak Ridge National Laboratory (Schultz *et al.*, 2014 ▸). The crystal mounting, data collection strategies and integration schemes were the same as in our previous study (Purevjav *et al.*, 2018 ▸). The crystal was measured in 17 different orientations for 2 d at 100 K. The proton beam power was 1.4 MW. For the structural analysis, we used 707 independent reflections covering the *d*-spacing range down to *d*_min_ = 0.50 Å; all these reflections satisfied the *I* > 3*σI* criteria.

### 2.3. Refinement of structural parameters   

The *hkl* reflection intensity dataset was analyzed using *General Structure Analysis System* (*GSAS*) software (Larson & Von Dreele, 2004 ▸). The initial structural parameters were taken from our previous powder neutron diffraction results (Tomioka *et al.*, 2016 ▸), though we did not use any constraints. First, the structural model was fit without D to obtain tentative structural parameters of Mg, Si and O. Using these parameters, we constructed the difference Fourier map to show the sites of D, which was located at the maximum nuclear density at 7.10 fm Å^−3^ between two adjacent layers of MgO_6_ octahedra. As discussed later, its coordinates were consistent with those of the tilted O—D dipole structure model [Fig. 1 ▸(*b*)]. After selecting this model, we refined the full structural parameters, including the D sites. The *wR*(*F*) and *R*(*F*) values obtained after full refinement at *d*_min_ = 0.50 Å were 5.3 and 6.1%, respectively. Additional series of refinements with *d*_min_ = 0.55, 0.60, 0.65 and 0.70 Å were conducted separately to evaluate the stability of cation occupancies (Fig. S2). Then, it was proved that the cation occupancies of DHMS phase E were stable for all these different *d*_min_ datasets.

## 3. Results and discussion   

**Figure 2:**
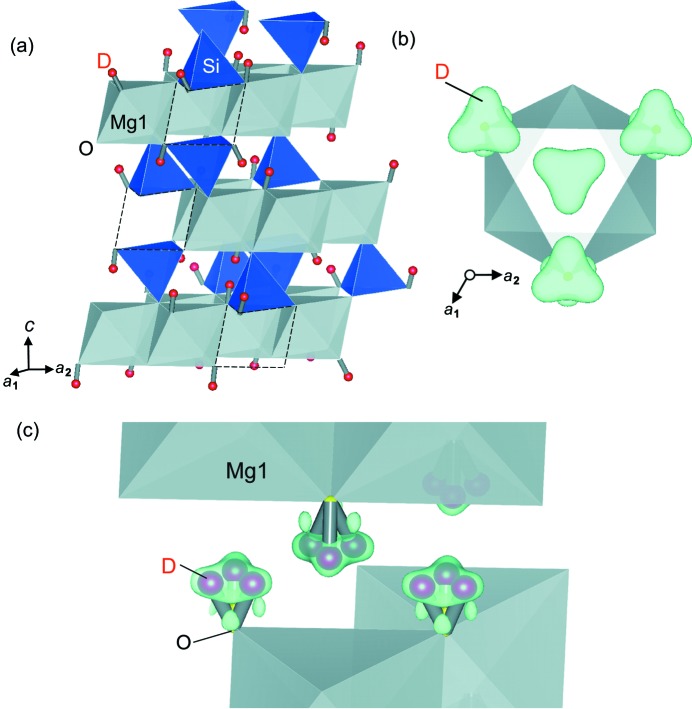
Refined structure of DHMS phase E. (*a*) Framework structure along with tilted O—D dipoles. No cation was found at the Mg2 site. Vacancies of MgO_6_ octahedra (dashed area) are coupled with two adjacent SiO_4_ tetrahedra crosslinking the neighboring layers. The proposed locations of D^+^ are shown as red spheres. (*b*) Difference Fourier map projected from the *c* axis orientation. The locations of D^+^ were determined along with refininement of the site occupancy factor to the corresponding nuclear density distributions. There are three equivalent sites of D^+^ around each O^2−^ ion. (*c*) Geometry of chemical bonding in the interlayer space O—D⋯O. Because of the site symmetry, three equivalent sites of D^+^ are observed simultaneously around each O^2−^, while one of them is actually filled by D^+^.

Table 1 ▸ shows the refined structural parameters at *d*_min_ = 0.50 Å. Fig. 2 ▸ shows the structure of the DHMS phase E. D^+^ is located between the MgO_6_ octahedral layers [Fig. 2 ▸(*a*)]. The nuclear density of D in the difference Fourier map forms a triangular shape [Fig. 2 ▸(*b*)], indicating that there are three equivalent sites of D (Wyckoff site at 18*h*) around each oxygen anion. Therefore, the O—D covalent bond is not along the direction normal to the octahedral layers, but towards the three neighboring O^2−^ ions of the adjacent MgO_6_ layer [Figs. 2 ▸(*b*) and 2 ▸(*c*)]. The tilted O—D dipole model is thus the suitable structural model. The O—D covalent bond distance is 0.817 (3) Å, which is identical to our previous powder diffraction results (Tomioka *et al.*, 2016 ▸). The O⋯D hydrogen bond distance is 2.088 (3) Å. The bonding angle of O—D⋯O is 163.3 (3)°, indicating that near-straight hydrogen bonding occurs between D^+^ and one of the three nearest-neighbor O^2−^ ions.

**Figure 3:**
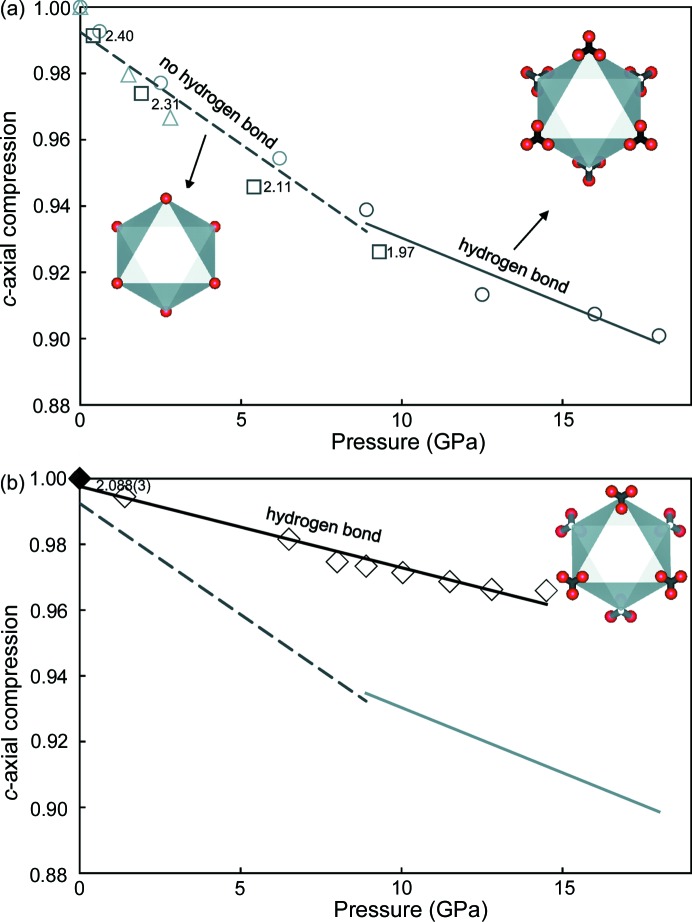
Compressibility along the *c* axis (*c*/*c*_0_) of brucite and DHMS phase E. (*a*) *c*/*c*_0_ of brucite reported by Nagai *et al.*, 2000 ▸ (circles); Okuchi *et al.*, 2014 ▸ (triangles); and Parise *et al.*, 1994 ▸ (squares). The solid and broken lines are linear fits of these data points at lower and higher pressures, respectively. The inset numbers show the distance D⋯O across the interlayer space at room temperature, obtained from Okuchi *et al.* (2014 ▸), and Parise *et al.* (1994 ▸). The distance monotonically decreases with increasing pressure to form a hydrogen bonding interlayer, which makes the structure distinctly harder. The inset figures show the D sites of brucite at ambient and high pressures, respectively. (*b*) *c*/*c*_0_ of DHMS phase E reported by Shieh *et al.*, 2000 ▸ (diamonds). The *c* axis of DHMS phase E around ambient pressure is already less compressible than that of brucite at high pressures. The inset number shows the distance D⋯O, determined in present study. The inset figure shows the D sites of DHMS phase E.

The structure of deuterated DHMS phase E at ambient pressure is close to that of deuterated brucite (magnesium deuteroxide) at high pressures (Okuchi *et al.*, 2014 ▸; Parise *et al.*, 1994 ▸). Both structures possess three-split D sites, clearly supporting the existence of interlayer hydrogen bonding in their structures (insets of Fig. 3 ▸). The interlayer distance between adjacent oxygen anions (O—D⋯O) in brucite is 3.22 Å at ambient pressure, which is too large for hydrogen bonding; on the other hand, the distance decreases to 2.88 Å at a pressure of 8.9 GPa, thereby enabling hydrogen bonding. In addition, the O—D⋯O angle of brucite is 148° at ambient pressure, which is too small for hydrogen bonding; however, this angle increases to 156° at 9.3 GPa, which again is consistent with the occurrence of hydrogen bonding. The *c* axis of brucite thus becomes distinctly less compressible at high pressures, indicating that its framework structure becomes harder with interlayer hydrogen bonding [Fig. 3 ▸(*a*)]. On the other hand, the distance of O—D⋯O in DHMS phase E is already 2.880 (1) Å at ambient pressure, which is suitable for hydrogen bonding of moderate strength. The O—D⋯O angle of 163° is also consistent with the occurrence of hydrogen bonding. Hence, hydrogen bonding occurs in DHMS phase E with a distance of 2.088 (3) Å at ambient pressure. It was previously reported that the *c* axis of DHMS phase E at ambient pressure is less compressible than that of brucite at high pressure, suggesting that interlayer hydrogen bonding already plays a role in hardening its framework structure [Fig. 3 ▸(*b*)]. Thus, we expect that interlayer hydrogen bonding in DHMS phase E becomes much stronger at high-pressure conditions inside deep Earth.

The presently determined O—D⋯O distance of DHMS phase E was more than 0.1 Å shorter than the value reported by Shieh *et al.* (2000 ▸), who suggested it exhibited weak hydrogen bonding. The reason for this difference is that Shieh *et al.* used the relation of the OH stretching frequencies versus O⋯O bond distances, where the relation had few data points, especially in the high-frequency range. Thus, such qualitative information is inaccurate for discussing the strength of hydrogen bonding in DHMS phase E.

Thermogravimetry analyses [Fig. 1 ▸(*a*)] demonstrated that the dehydration of DHMS phase E at ambient pressure occurs at a much higher temperature than that of brucite. At high pressure, the dehydration temperature of brucite increases, reaching 1550 K at 15 GPa (Johnson & Walker, 1993 ▸); this demonstrates the important role that hydrogen bonding plays in the stability of the structure against heat. Furthermore, the pressure-enhanced hydrogen bonding in DHMS phase E should act to increase the dehydration temperature. We conclude that strong hydrogen bonding is the most important factor for the high-temperature stability of DHMS phase E in deep Earth. Its high-temperature stability limit has not yet been accurately determined; nevertheless, it is stable to at least 1573 K at a pressure of 15 GPa (Frost, 1999 ▸). We expect that hydrogen bonding also plays a universal role in enhancing the stability of various hydrous minerals in deep Earth, and we will seek to verify this in our future research.

It has been reported that DHMS phase E incorporates a variable amount of hydrogen (Frost, 1999 ▸; Tomioka *et al.*, 2016 ▸), thereby allowing flexible cation substitution, including hydrogen as one of the exchangeable species. We found a considerable number of Mg^2+^ vacancies at the Mg1 site, but no cations at the previously proposed Mg2 site. We found that the Si^4+^ and D^+^ sites were very close to each other. Furthermore, we considered the structural relation between brucite and DHMS phase E, as well as a full disordering of cations, as required by the crystallographic symmetry. It was concluded that multiple D^+^ ions in the interlayer space were simultaneously exchanged with the Si^4+^ ions that connect the neighboring layers, together with the generation of Mg^2+^ vacancies inside the MgO_6_ octahedral layers. By comparing the refined chemical formula of the DHMS phase E crystal (Mg_2.28_Si_1.32_D_2.15_O_6_) with that of brucite (Mg_3_Si_0_D_6_O_6_), we found that the exchange mechanism of four possible models have the DHMS phase E structure from brucite, while maintaining the cation charge balances. We calculated the balances in occupancies of Mg and D for these models and compared them with those of our refinement result (see Fig. S3). We found that most plausible model is
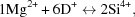

*i.e.* one Mg^2+^ in the MgO_6_ octahedral layer and six D^+^ in the interlayer space are exchanged with two Si^4+^ at the top and bottom of the Mg^2+^ vacancy [Fig. 2 ▸(*a*)]. The cation-to-cation distance is too short for the SiO_4_ tetrahedron and MgO_6_ octahedron to share faces; consequently, Mg^2+^ must be removed to introduce two SiO_4_ tetrahedra which share their faces with the same Mg1 site. These SiO_4_ tetrahedra have a deformed geometry, with an Si—O distance of 1.666 (3) Å along the *c* axis and 1.8322 (9) Å along the other directions. The O^2−^ bonded to Si^4+^ with a shorter distance along the *c* axis does not possess a D^+^ ion, thereby avoiding repulsion between Si^4+^ and D^+^. Two of the other three O^2−^ bonded to Si^4+^ with a longer distance possess D^+^ to form two tilted O—D dipoles towards their interlayer hydrogen bonding directions. Thus, the hydrogen capacity in the DHMS phase E structure is eventually controlled by the exchanged amount of Si^4+^, while maintaining site disordering of all cations.

## 4. Conclusions   

We analyzed the chemical bonding geometry around hydrogen in the framework structure of phase E, which is representative of the dense hydrous magnesium silicate (DHMS) minerals that retain hydrogen within deep Earth. A single crystal of deuterated DHMS phase E was synthesized at high pressure and temperature and subsequently analyzed using TOF neutron Laue diffraction. The nuclear density distribution of D^+^ in the DHMS phase E framework structure at 100 K was obtained with a high spatial resolution of *d*_min_ = 0.50 Å. It was found that, within the layered structure of DHMS phase E, the O—D dipole was tilted from the direction normal to the MgO_6_ octahedral layers due to the occurrence of interlayer hydrogen bonding to one of the neighboring O^2−^ ions. This geometry of the hydrogen bonds was similar to that of compressed brucite at high pressures. The hydrogen bond length of DHMS phase E at ambient pressure was comparable with that of brucite at high pressure. By referring to compressibility studies on DHMS phase E and brucite, which have similar structures made of MgO_6_ octahedral layers and interlayer spaces, we conclude that hydrogen bonding in these minerals plays a crucial role in increasing their dehydration temperatures. However, the role of hydrogen bonding is more significant in DHMS phase E than in brucite. We propose that cation exchange of Mg^2+^, D^+^ and Si^4+^ approximately −1:−6:+2 (molar ratio) occurs within the DHMS phase E structure while retaining full disordering of the cation sites.

## Supplementary Material

Crystal structure: contains datablock(s) PHASE-E-08-20190409_publ. DOI: 10.1107/S2052252520003036/fs5179sup1.cif

Structure factors: contains datablock(s) PHASE-E-08-20190409_publ. DOI: 10.1107/S2052252520003036/fs5179sup2.hkl

Supporting information file. DOI: 10.1107/S2052252520003036/fs5179sup3.pdf

CCDC reference: 1990294

## Figures and Tables

**Table 1:** Refined structural parameters at *d*_min_ = 0.50 Å The lattice parameters are *a* = 2.9647 (4) and *c* = 13.8892 (3) Å, determined by single-crystal neutron diffraction at 100 K.

Wyckoff sites	Atoms	Atomic coordinates			Occupancies	Debye–Waller factors				
		*x*	*y*	*z*		*U*_iso_ †	*U*_11_ ‡ = *U*_22_	*U*_33_	*U*_12_	*U*_13_ = *U*_23_
3*c*	Mg1	0	0	0	0.761 (4)		0.01693 (3)	0.0150 (5)	0.0085 (2)	0
6*c*	Si	0	0	0.1301 (2)	0.220 (3)		0.0110 (6)	0.008 (1)	0.0055 (3)	0
6*c*	O1	0.6667	0.3333	0.08328 (4)	1		0.0199 (2)	0.0200 (3)	0.00100 (1)	0
18*h*	D	−0.0668 (5)	0.0668 (5)	0.1965(2)	0.119 (1)	0.0358 (8)				

† Isotropic.

‡ Anisotropic.

## References

[bb1] Frost, D. J. (1999). *Geo. Soc.*, **6**, 283–296.

[bb2] Iizuka-Oku, R., Yagi, T., Gotou, H., Okuchi, T., Hattori, T. & Sano-Furukawa, A. (2017). *Nat. Commun.* **8**, 14096. 10.1038/ncomms14096 PMC5241797 28082735

[bb3] Johnson, M. C. & Walker, D. (1993). *Am. Mineral.* **78**, 271–284.

[bb4] Kanzaki, M. (1991). *Phys. Earth Planet. Inter.* **66**, 307–312.

[bb5] Kawakatsu, H. & Watada, S. (2007). *Science*, **316**, 1468–1471. 10.1126/science.1140855 17556582

[bb6] Kudoh, Y., Finger, L. W., Hazen, R. M., Prewitt, C. T., Kanzaki, M. & Veblen, D. R. (1993). *Phys. Chem. Miner.* **19**, 357–360.

[bb7] Larson, A. C. & Von Dreele, R. B. (2004). *General Structure Analysis System (GSAS).* Los Alamos National Laboratory, New Mexico, USA.

[bb8] Momma, K. & Izumi, F. (2011). *J. Appl. Cryst.* **44**, 1272–1276.

[bb9] Nagai, T., Hattori, T. & Yamanaka, T. (2000). *Am. Mineral.* **85**, 760–764.

[bb10] Nishi, M., Irifune, T., Tsuchiya, J., Tange, Y., Nishihara, Y., Fujino, K. & Higo, Y. (2014). *Nat. Geosci.* **7**, 224–227.

[bb11] Ohtani, E. (2015). *Chem. Geol.* **418**, 6–15.

[bb12] Ohtani, E., Mizobata, H. & Yurimoto, H. (2000). *Phys. Chem. Miner.* **27**, 533–544.

[bb13] Okuchi, T. (1997). *Science*, **278**, 1781–1784. 10.1126/science.278.5344.1781 9388177

[bb14] Okuchi, T., Purevjav, N., Tomioka, N., Lin, J. F., Kuribayashi, T., Schoneveld, L., Hwang, H., Sakamoto, N., Kawasaki, N. & Yurimoto, H. (2015). *Am. Mineral.* **100**, 1483–1492.

[bb15] Okuchi, T., Tomioka, N., Purevjav, N., Abe, J., Harjo, S. & Gong, W. (2014). *High. Press. Res.* **34**, 273–280.

[bb16] Parise, J. B., Leinenweber, K., Weidner, D. J., Tan, K. & Vondreele, R. B. (1994). *Am. Mineral.* **79**, 193–196.

[bb17] Purevjav, N., Okuchi, T., Tomioka, N., Abe, J. & Harjo, S. (2014). *Geophys. Res. Lett.* **41**, 6718–6724.

[bb18] Purevjav, N., Okuchi, T., Tomioka, N., Wang, X. P. & Hoffmann, C. (2016). *Sci. Rep.* **6**, 34988. 10.1038/srep34988 PMC5057097 27725749

[bb19] Purevjav, N., Okuchi, T., Wang, X., Hoffmann, C. & Tomioka, N. (2018). *Acta Cryst.* B**74**, 115–120.

[bb20] Sano-Furukawa, A., Hattori, T., Komatsu, K., Kagi, H., Nagai, T., Molaison, J. M., Dos Santos, A. M. & Tulk, C. A. (2018). *Sci. Rep.* **8**, 15520. 10.1038/s41598-018-33598-2 PMC6195538 30341340

[bb21] Schultz, A. J., Jørgensen, M. R. V., Wang, X., Mikkelson, R. L., Mikkelson, D. J., Lynch, V. E., Peterson, P. F., Green, M. L. & Hoffmann, C. M. (2014). *J. Appl. Cryst.* **47**, 915–921.

[bb22] Shieh, S. R., Mao, H. K., Konzett, J. & Hemley, R. J. (2000). *Am. Mineral.* **85**, 765–769.

[bb23] Thompson, A. B. (1992). *Nature*, **358**, 295–302.

[bb24] Tomioka, N., Okuchi, T., Purevjav, N., Abe, J. & Harjo, S. (2016). *Phys. Chem. Miner.* **43**, 267–275.

