# Supplementary material for: Strong hydrogen bonding in a dense hydrous magnesium silicate discovered by neutron Laue diffraction
Source: IUCrJ. 2020 Apr 2;7(Pt 3):370–4. doi: 10.1107/S2052252520003036 (PMC7201286; doi:10.1107/S2052252520003036)
Supplement: Supplementary file 3 [file m-07-00370-sup3.pdf]

IUCrJ

Volume 7 (2020)

Supporting information for article:

Strong hydrogen bonding in a dense hydrous magnesium silicate discovered by neutron Laue diffraction

Narangoo Purevjav, Takuo Okuchi and Christina Hoffmann

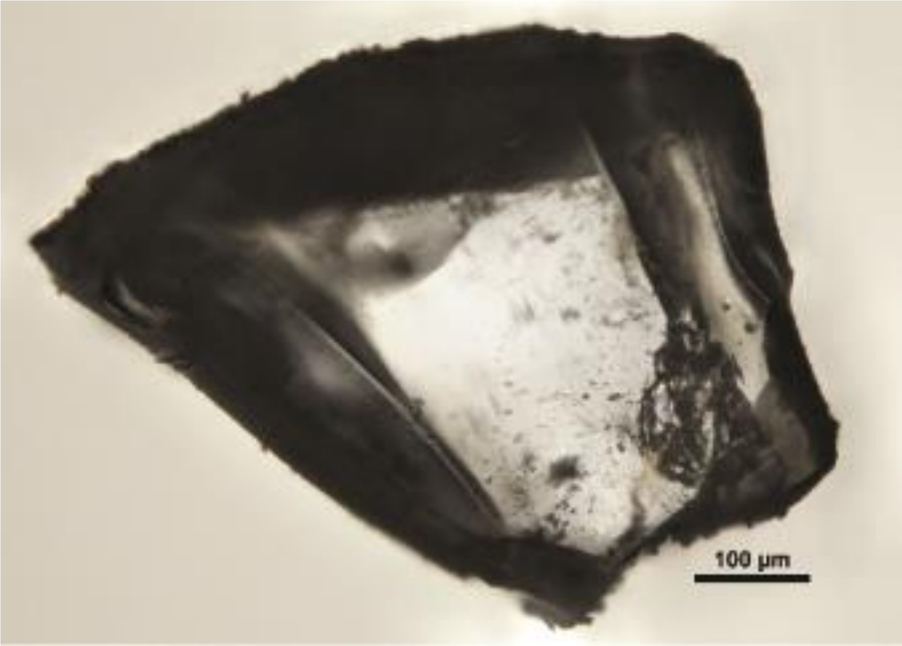

Figure S1 Recovered single crystal of deuterated dense hydrous magnesium silicate (DHMS) phase E, measured by time-of-flight (TOF) single-crystal neutron Laue diffraction.

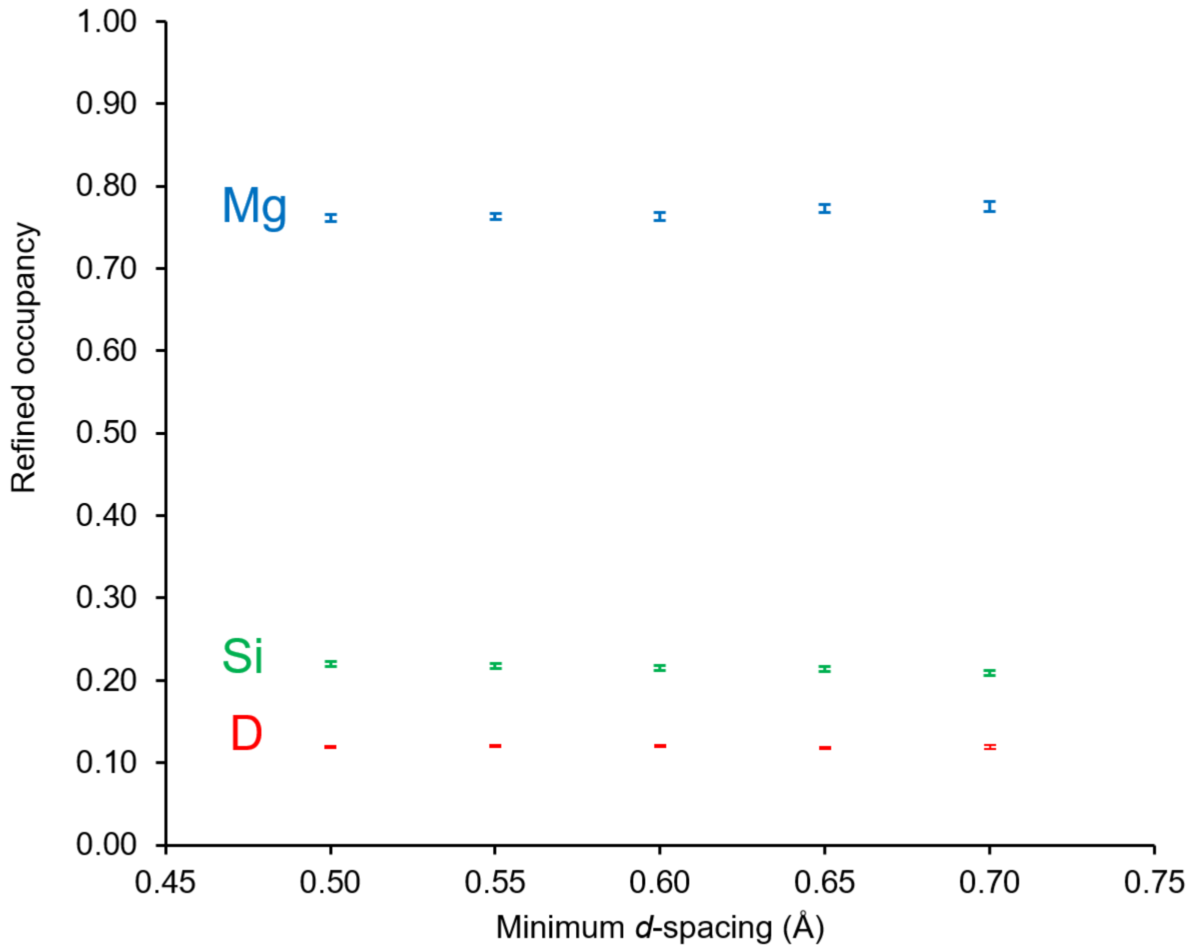

Figure S2 Refined cation occupancies as a function of minimum *d*–spacing. Each symbol indicates the refined occupancies along with the standard deviations. The numbers of reflections [$I > 3\sigma(I)$] are as follows: 707 at d_{min} = 0.50 Å, 652 at d_{min} = 0.55 Å, 580 at d_{min} = 0.60 Å, 488 at d_{min} = 0.65 Å, 424 at d_{min} = 0.70 Å.

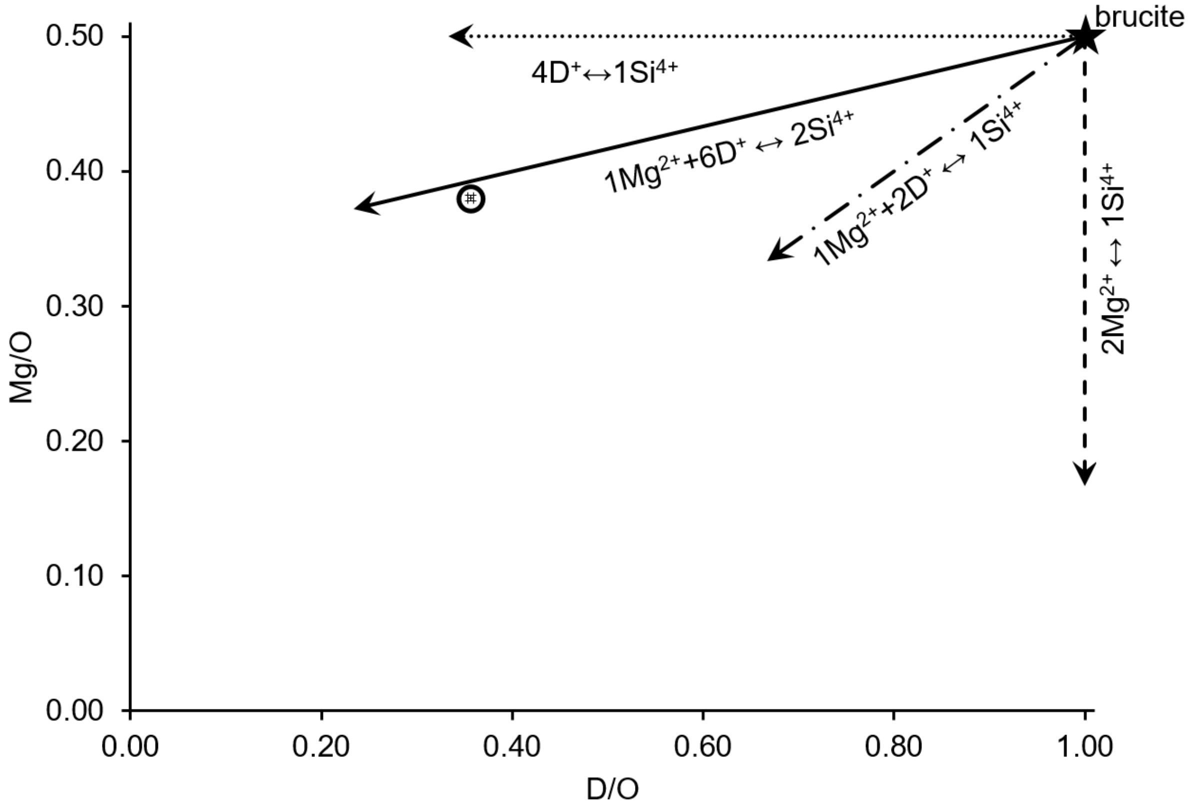

Figure S3 Mg/O and D/O atomic ratio of the four proposed models (lines) and structure refinement result (open circle).
